# Polyacrylic Surfactant-Enabled Engineering of Co_3_O_4_ Electrodes for Enhanced Asymmetric Supercapacitor Performance

**DOI:** 10.3390/ma18122916

**Published:** 2025-06-19

**Authors:** Rutuja U. Amate, Pritam J. Morankar, Mrunal K. Bhosale, Aviraj M. Teli, Sonali A. Beknalkar, Chan-Wook Jeon

**Affiliations:** 1 School of Chemical Engineering, Yeungnam University, 280 Daehak-ro, Gyeongsan 712-749, Republic of Korea; rutu.nanoworld@gmail.com (R.U.A.); pritam.nanoworld@gmail.com (P.J.M.); mrunal.snst.1@gmail.com (M.K.B.); 2 Division of Electronics and Electrical Engineering, Dongguk University-Seoul, Seoul 04620, Republic of Korea; avteli.teli@gmail.com (A.M.T.); sonalibeknalkar@gmail.com (S.A.B.)

**Keywords:** PAA-modified CO, electrodeposition, nanosheets, charge storage, stability, asymmetric pouch-type supercapacitor

## Abstract

In this work, we report a facile and tunable electrodeposition approach for engineering polyacrylic acid (PAA)-modified Co_3_O_4_ electrodes on nickel foam for high-performance asymmetric pouch-type supercapacitors. By systematically varying the PAA concentration (0.5 wt %, 1 wt %, and 1.5 wt %), we demonstrate that the CO-1 sample (1 wt % PAA) exhibited the most optimized structure and electrochemical behavior. The CO-1 electrode delivered a remarkable areal capacitance of 3467 mF/cm^2^ at 30 mA/cm^2^, attributed to its interconnected nanosheet morphology, enhanced ion diffusion, and reversible Co^2+^/Co^3+^/Co^4+^ redox transitions. Electrochemical impedance spectroscopy confirmed low internal resistance (0.4267 Ω), while kinetic analysis revealed a dominant diffusion-controlled charge storage contribution of 91.7%. To evaluate practical applicability, an asymmetric pouch-type supercapacitor device was assembled using CO-1 as the positive electrode and activated carbon as the negative electrode. The device operated efficiently within a 1.6 V window, achieving an impressive areal capacitance of 157 mF/cm^2^, an energy density of 0.056 mWh/cm^2^, a power density of 1.9 mW/cm^2^, and excellent cycling stability. This study underscores the critical role of polymer-assisted growth in tailoring electrode architecture and provides a promising route for integrating cost-effective and scalable supercapacitor devices into next-generation energy storage technologies.

## 1. Introduction

In response to the escalating global energy demand and the depletion of fossil fuel reserves, the pursuit of efficient and sustainable energy storage technologies has become a paramount global challenge. Among the various emerging solutions, electrochemical energy storage devices, particularly supercapacitors, have garnered considerable interest due to their unique ability to bridge the gap between traditional capacitors and batteries. Supercapacitors are characterized by their rapid charge–discharge capability, high power density, excellent reversibility, and long-term operational stability, making them highly suitable for next-generation portable electronics, electric vehicles, and storage applications [1,2,3]. Supercapacitors can be broadly categorized into electric double-layer capacitors (EDLCs) and pseudocapacitors, based on their charge storage mechanisms. EDLCs store energy through the non-Faradaic process of electrostatic ion accumulation at the electrode–electrolyte interface, whereas pseudocapacitors rely on fast and reversible Faradaic redox reactions involving electron transfer between the electrode surface and electrolyte ions [4,5,6,7]. Compared to EDLCs, pseudocapacitors generally offer higher specific capacitance and energy density, although often at the cost of slightly reduced power performance.

Transition metal oxides (TMOs), such as MnO_2_, NiO, and Co_3_O_4_, have emerged as promising pseudocapacitive materials due to their multiple oxidation states, high theoretical capacitance, and intrinsic redox activity. Among these, cobalt oxide (Co_3_O_4_) has received particular attention owing to its rich redox chemistry, spinel crystal structure, and a high theoretical capacitance of approximately 3560 F/g. Co_3_O_4_ features a mixed-valence structure (Co^2+^ and Co^3+^), which facilitates reversible redox reactions in both aqueous and non-aqueous electrolytes, making it a strong candidate for advanced supercapacitor electrodes [8,9,10]. Beyond its theoretical advantages, Co_3_O_4_ exhibits excellent chemical and thermal stability, low cost, and favorable electrochemical reversibility. However, the electrochemical performance of Co_3_O_4_ is critically influenced by its physical and structural characteristics, including crystallinity, surface area, pore distribution, and surface morphology. These factors govern ion diffusion and charge transfer kinetics during operation. To overcome inherent limitations, such as poor electrical conductivity and ion diffusion resistance in bulk Co_3_O_4_, significant efforts have been devoted to nanostructuring and morphological engineering. Strategies such as the fabrication of nanosheets, nanowires, nanorods, and hollow spheres have demonstrated notable improvements in electrochemical performance by reducing diffusion paths and increasing the electroactive surface area [11,12]. Such architectures offer short diffusion pathways, high surface-to-volume ratios, and structural robustness, factors that collectively enhance electrochemical performance. A number of studies have explored the morphological tailoring of Co_3_O_4_ using various synthesis routes. Wang et al. synthesized porous Co_3_O_4_ nanowires by annealing cobalt-carbonate-hydroxide precursors, achieving a specific capacitance of 240 F/g and excellent cycling stability [13]. In another approach, Wang et al. demonstrated enhanced oxygen evolution reaction (OER) activity and high capacitance (978 F/g) in NaBH_4_-treated mesoporous Co_3_O_4_ nanowires enriched with oxygen vacancies, attributed to improved electronic conductivity from defect engineering [14]. Duan et al. reported a hierarchically porous Co_3_O_4_ film using polystyrene templating, yielding a high specific capacitance of 454 F/g and excellent stability, which was attributed to optimized ion/electron transport [15]. Yang et al. developed a nanowire network of Co_3_O_4_ directly grown on carbon fiber paper that delivered 1124 F/g at 25.34 A/g and retained 94% of capacitance, underscoring the mechanical robustness and redox efficiency of 3D frameworks [16]. While these methods have yielded promising performance metrics, they often suffer from limitations, such as complex multi-step processing, high-temperature treatment, use of hazardous reagents, or limited scalability, factors that impede their integration into practical, flexible, and large-scale supercapacitor devices [17,18].

In contrast, electrodeposition has emerged as a simple, cost-effective, and scalable approach for fabricating Co_3_O_4_ thin films and nanostructures directly on conductive substrates. This method offers precise control over deposition thickness, composition, and morphology at room temperature, without the need for post-synthetic annealing or complex chemical treatments. Despite its advantages, the electrodeposited Co_3_O_4_ films often suffer from poor microstructural control, leading to dense, nonporous morphologies with limited ion accessibility and active surface exposure. This constraint can be mitigated through the strategic introduction of structure-directing agents, such as surfactants, during the electrodeposition process. The surfactants, also known as surface-directing agents, have been widely employed to manipulate growth kinetics and morphology. Among various surfactants, polyacrylic acid (PAA) is particularly appealing due to its unique chemical and physicochemical properties. PAA is a water-soluble polyelectrolyte with carboxylic acid functional groups that can chelate metal ions, modulate the local pH, and influence nucleation and crystal growth kinetics. Its biocompatibility, tunable viscosity, and pH-responsiveness make it a versatile candidate for directing the morphology and surface texture of metal oxide nanostructures. In the context of Co_3_O_4_ synthesis, however, PAA remains largely underexplored, especially in electrodeposition processes where its interaction with cobalt precursors can lead to previously unattainable morphologies. In addition to its ability to influence crystal nucleation, PAA can also act as a soft template, facilitating the formation of nanosheet-like or porous structures with improved electrolyte accessibility. Unlike rigid templates or inorganic surfactants, PAA provides dynamic coordination, enabling more uniform and controllable growth. This coordination can yield films with increased porosity, well-exposed electroactive sites, and improved ion diffusion pathways, crucial features for high-performance pseudocapacitive behavior. Despite these theoretical advantages, a systematic investigation of how varying the PAA concentration affects the growth behavior, surface composition, and electrochemical performance of electrodeposited Co_3_O_4_ remains lacking [19,20,21].

Importantly, most prior works have not correlated the surfactant concentration with elemental composition, post-cycling morphology retention, or real-device performance, limiting their practical applicability. In light of these gaps, the present study aims to address several critical issues. We report the electrochemical deposition of Co_3_O_4_ nanostructures on nickel foam using a PAA-assisted strategy, with systematic variation in the surfactant concentration (0.5%, 1%, and 1.5%) to control morphology and optimize electrochemical behavior. The influence of PAA on the crystallographic features, surface composition, and cobalt/oxygen atomic ratios is investigated using physiochemical analyses. Electrochemical performance is evaluated using cyclic voltammetry (CV), galvanostatic charge–discharge (GCD), and electrochemical impedance spectroscopy (EIS). To demonstrate the real-world utility of the optimized material, an all-solid-state asymmetric pouch-type supercapacitor device (APSD) is fabricated using optimized electrodes.

## 2. Experimental Section

### Electrodeposition-Based Fabrication of PAA-Modified CO Electrodes

**Figure 1:**
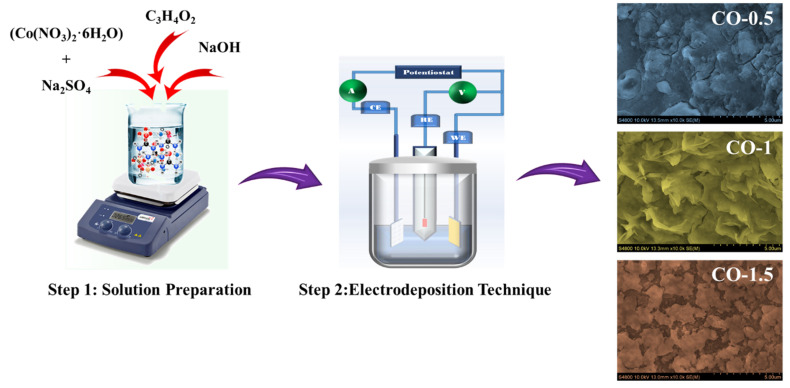
Schematic illustration of the PAA-assisted electrodeposition process for synthesizing CO electrodes.

CO electrodes were fabricated on pre-cleaned nickel foam substrates through a controlled electrodeposition process designed to enhance electrochemical performance by incorporating varying concentrations of PAA. The deposition electrolyte was prepared by dissolving 0.15 M cobalt nitrate hexahydrate (Co(NO_3_)_2_·6H_2_O), 0.15 M sodium sulfate (Na_2_SO_4_), and 0.04 M sodium hydroxide (NaOH) in 100 mL of deionized (DI) water, followed by continuous magnetic stirring to ensure uniform mixing. To assess the influence of PAA, different weight percentages (0.5 wt %, 1.0 wt %, and 1.5 wt %) were incorporated into the electrolyte prior to deposition. Electrodeposition was conducted at room temperature using a standard three-electrode configuration: the PAA-treated nickel foam served as the working electrode, a platinum wire as the counter electrode, and a saturated Ag/AgCl electrode as the reference. The films were deposited within a potential window of −1.2 V to +1.0 V versus Ag/AgCl, for 25 electrodeposition cycles. After the deposition, all samples were thoroughly rinsed with DI water, dried at 80 °C overnight, and subsequently annealed in air at 400 °C for 2 h to enhance crystallinity and adhesion. The fabricated electrodes were designated as CO (without PAA), CO-0.5 (0.5 wt % PAA), CO-1.0 (1.0 wt % PAA), and CO-1.5 (1.5 wt % PAA). Schematic illustration of the PAA-assisted electrodeposition process for synthesizing CO electrodes were depicted in Figure 1.

## 3. Sample Characterization and Electrochemical Measurements

The phase composition and crystallinity of the CO electrodes were examined via XRD (PAN-analytical, Cu-Kα source, Malvern Panalytical, Malvern, UK), allowing clear identification of crystalline phases and structural integrity. Surface architecture and elemental distribution were studied using FE-SEM (S4800, HITACHI, Tokyo, Japan) combined with EDS (energy-dispersive X-ray spectroscopy) analysis. Prior to imaging, a thin platinum layer was sputtered onto the samples to minimize charging effects. FE-SEM images revealed detailed surface textures, while EDS confirmed the elemental presence and dispersion. For insights into surface chemistry and oxidation states, X-ray photoelectron spectroscopy (XPS; K-Alpha, Thermo Scientific, Oxford, UK) was employed, enabling evaluation of elemental valency. Electrochemical assessments were carried out using a Biologic WBCS3000 battery cycler (BioLogic, Seyssinet-Pariset, France) in a three-electrode setup, where the CO-1 electrode acted as the working electrode, with platinum and Ag/AgCl as the counter and reference electrodes, respectively. A 2 M KOH solution served as the electrolyte to probe capacitance, charge–discharge characteristics, and cycling stability.

## 4. Results and Discussion

### 4.1. XRD Elucidation

**Figure 2:**
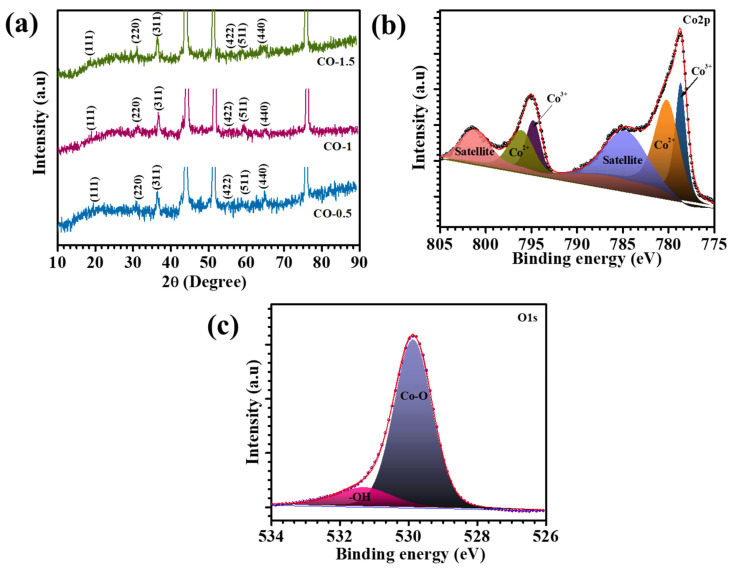
(**a**) XRD pattern of CO electrodes, and high-resolution spectra of (**b**) Co2p and (**c**) O1s spectra of CO-1 electrode.

XRD analysis was conducted to comprehensively investigate the structural integrity, crystallinity, and phase composition of the synthesized nanocomposites. Figure 2a presents the XRD patterns of the CO-0.5, CO-1, and CO-1.5 samples, providing insight into their crystalline structures. The CO-1 composite exhibited well-defined diffraction peaks at 18.9°, 31.2°, 36.7°, 55.5°, 59.3°, and 64.2°, corresponding to the (111), (220), (311), (422), (511), and (440) crystal planes, respectively. These peaks are in excellent agreement with the standard diffraction data from JCPDS Card No. 00-042-1467, confirming the formation of phase-pure Co_3_O_4_ with a characteristic cubic structure [22]. The XRD profiles of CO-0.5 and CO-1.5 composites showed similar peak positions, indicating the preservation of the same crystalline phase across all samples. Importantly, the incorporation of PAA at varying concentrations did not alter the crystallinity of the materials, as evidenced by the consistent peak intensities and sharpness. This observation confirmed that PAA did not disrupt the crystallization process of Co_3_O_4_. A minor shift in peak positions was observed with increasing PAA content, suggesting subtle variations in the lattice parameters. This shift was likely induced by the presence of PAA, which may influence the packing density and induce a more compact crystal structure. Despite these shifts, the core crystallographic features remained unchanged. Overall, the XRD analysis validated the successful synthesis of single-phase, highly crystalline Co_3_O_4_. The preservation of crystallinity and phase purity following PAA incorporation underscores the structural stability of the material, a crucial factor for its potential application in energy storage systems where crystallinity plays a pivotal role in enhancing catalytic activity and long-term performance.

### 4.2. XPS Analysis

The surface elemental composition and oxidation states of cobalt (Co) and oxygen (O) in the synthesized Co_3_O_4_ material were systematically investigated through XPS. High-resolution spectra were acquired for the Co2p and O1s core levels to gain insights into the electronic environment and chemical states of these elements. Specifically, the Co2p_3/2_ (Figure 2b) displayed two main peaks located at binding energies of approximately 778.6 eV and 780.1 eV, which were attributed to Co^3+^ and Co^2+^ oxidation states, respectively. Furthermore, the Co2p_1/2_ region exhibited peaks centered at 794.7 eV and 796.1 eV, which were likewise assigned to Co^3+^ and Co^2+^, respectively [23,24]. In addition to these primary peaks, two characteristic satellite peaks were observed at 784.8 eV and 801.3 eV, further providing evidence of multiple splitting and final state effects typically associated with transition metal oxides. The high-resolution O 1s spectrum presented in Figure 2c further corroborates the chemical state of the oxygen species present in the Co_3_O_4_ lattice. A dominant peak located at 529.8 eV corresponded to lattice oxygen bound within metal–oxygen (Co–O) frameworks, signifying the formation of Co–O bonds typical of the Co_3_O_4_ spinel structure [25]. An additional shoulder peak appeared at 531.2 eV, which was ascribed to surface-adsorbed hydroxyl species (–OH groups) [23]. Overall, the deconvoluted XPS spectra conclusively indicated that the Co_3_O_4_ material exhibited the expected mixed-valence state of cobalt ions (Co^2+^/Co^3+^) and contained both lattice-bound and surface-adsorbed oxygen species. These findings are crucial for understanding the surface reactivity, catalytic behavior, and redox properties of Co_3_O_4_ in various energy storage applications.

### 4.3. Morphological and Elemental Composition

**Figure 3:**
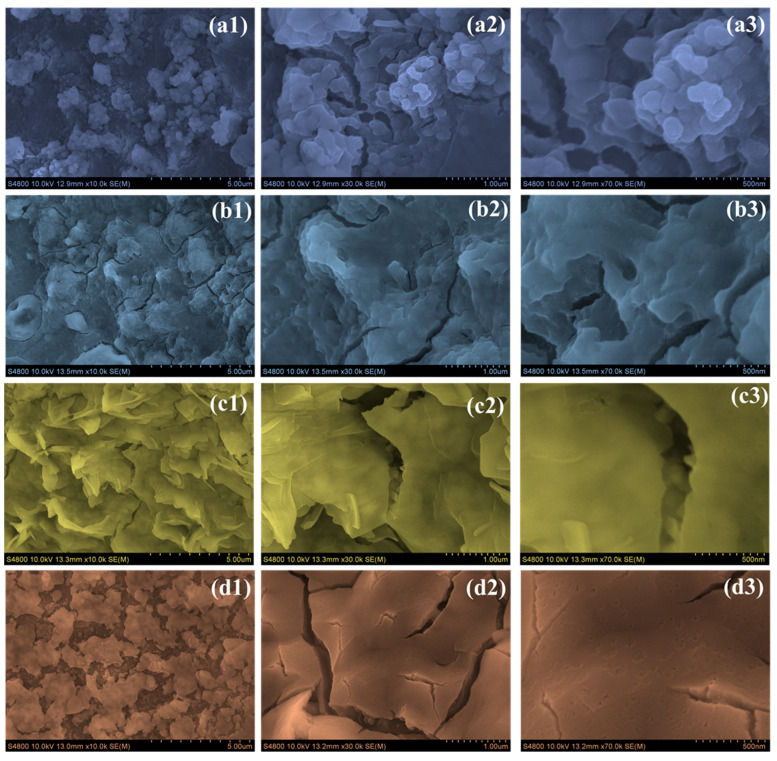
FE-SEM images of (**a1**–**a3**) CO, (**b1**–**b3**) CO-0.5, (**c1**–**c3**) CO-1, and (**d1**–**d3**) CO-1.5 samples at different magnifications.

The surface morphology of the CO electrodes showed a clear transformation with the introduction of PAA, as evident from the FE-SEM images at various magnifications (Figure 3(a1–a3)). In the case of the sample prepared without PAA (CO), the surface appeared densely packed with irregular, heavily agglomerated particles. The structure lacked noticeable porosity, and the grain boundaries were not well-defined. This compact and disordered arrangement is likely to restrict the active surface area and limit efficient ion transport during electrochemical reactions. When 0.5 wt % PAA was added, the CO-0.5 electrode exhibited a noticeable but modest change in surface structure (Figure 3(b1–b3)). The morphology began to shift slightly, developing a more flaky, layered texture with a few visible gaps. Compared to the densely packed surface of the CO electrode, this version appeared slightly more open and textured. This suggests that even a small amount of PAA influenced particle growth, although the overall structure remained relatively compact and may not yet provide optimal conditions for efficient ion transport [26]. The CO-1 electrode, which contains 1 wt % PAA, showed the most noticeable and beneficial change in morphology (Figure 3(c1–c3)). At all magnification levels, the surface was covered with thin nanosheets that stood upright and were evenly spaced. These nanosheets were well-connected, forming an open, porous network. This kind of structure increases the surface area and allows ions to move more easily in and out of the material. Compared to the earlier electrodes, there was no obvious clumping or irregularities—everything looked clean and well-organized. This structure is especially favorable for electrochemical applications because it improves ion movement and offers a more active surface area, which matches well with the better performance we observed in charge storage and cycling tests [27]. On the other hand, the CO-1.5 electrode (with 1.5 wt % PAA) seemed to suffer from having too much additive (Figure 3(d1–d3)). The surface looked more compact, with nanosheets piling on top of each other and starting to collapse. There were also visible cracks, which suggested that the film’s structure was weakened. It seems that too much PAA interfered with the film formation process, making the structure denser and less porous. As a result, it was harder for the electrolyte to get in, and there were fewer active sites for reactions, which can negatively impact the overall performance. From the surface features, it is clear that the CO-1 electrode offered the best balance between structure and function, making it the most suitable for electrochemical use [28].

**Figure 4:**
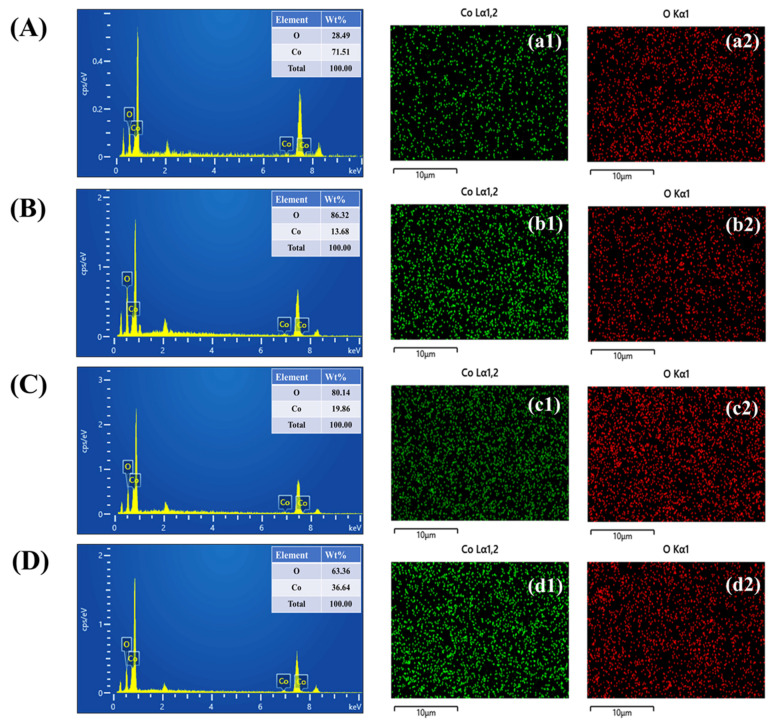
(**A**–**D**) EDS analysis and (**a1**–**d2**) elemental mapping of CO samples.

Elemental composition analysis of the PAA-modified CO electrodes, namely, the unmodified sample C0 and the CO-0.5, CO-1, and CO-1.5 variants, was carried out using EDS. The spectral profiles shown in Figure 4A–D clearly identify the presence of cobalt and oxygen in each case, indicating the successful incorporation of CO onto the nickel foam framework. Insets within Figure 4A–D provide the corresponding elemental weight percentage data. To further investigate the distribution of these elements, EDS elemental mapping was employed. The resulting images in Figure 4(a1–d2) demonstrate a well-dispersed and uniform allocation of cobalt and oxygen throughout the surface of all hydrothermally fabricated electrodes. This consistent elemental spread across all samples points to the structural uniformity and reliable synthesis of the electrode materials, underlining their potential effectiveness in electrochemical device applications.

## 5. Electrochemical Analysis

**Figure 5:**
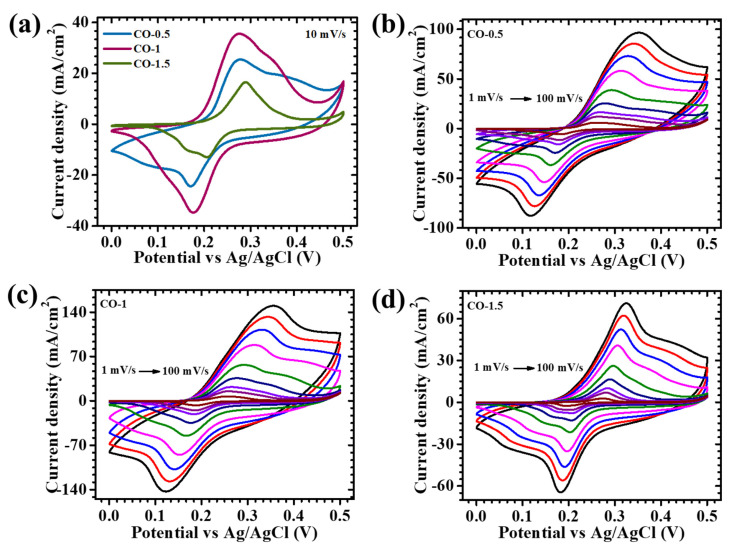
Cyclic voltammetry of (**a**) all CO electrodes at a scan rate of 10 mV/s, in a potential window of 0 to 0.5 V, and cyclic voltammetry of (**b**) CO-0.5, (**c**) CO-1, and (**d**) CO-1.5 samples at different scan rates (3–100 mV/s).

The electrochemical characteristics of CO electrodes synthesized with varying concentrations of PAA—0.5%, 1%, and 1.5%—were thoroughly examined using cyclic voltammetry (CV), galvanostatic charge–discharge (GCD), and electrochemical impedance spectroscopy (EIS) within a conventional three-electrode system, employing 2 M KOH as the electrolyte. Figure 5a presents the CV responses of the CO-0.5, CO-1, and CO-1.5 samples recorded at a scan rate of 10 mV/s across a potential range of 0 to 0.5 V. The incorporation of PAA exerted a notable influence on the redox activity and capacitive behavior of the CO electrodes, highlighting the surfactant’s crucial role in modulating material properties. This modulation was primarily attributed to PAA’s function as a structure-directing agent during synthesis, which governed nucleation and growth kinetics, ultimately affecting crystal morphology, surface roughness, and ion-accessible active sites. To assess the rate performance and reversibility of redox processes, CV measurements were further carried out at variable scan rates, ranging from 1 to 100 mV/s (Figure 5b–d). For reference, the corresponding electrochemical profile of pristine CO (synthesized without PAA) is provided in Figure S1a. All PAA-assisted electrodes displayed broad, well-defined redox peaks, centered around ~0.3 V (oxidation) and 0.1–0.2 V (reduction), characteristic of Faradaic transitions associated with the Co^2+^/Co^3+^ and Co^3+^/Co^4+^ redox couples [29]. The non-rectangular shape of the CV curves, along with distinct redox peaks, confirmed a pseudocapacitive charge storage mechanism, governed predominantly by reversible surface redox reactions rather than electrostatic double-layer capacitance. These features arose from the reversible intercalation and deintercalation of OH^−^ ions into the Co_3_O_4_ lattice, represented by the following reaction (1) [30]:

(1) $$Co_{3}O_{4}+H_{2}O+OH^{-}⇌\;3CoOOH+e^{-}$$

Among the tested samples, the CO-1 electrode exhibited the most favorable electrochemical performance, likely due to an optimal balance between particle dispersion, surface area, and structural uniformity. In contrast, lower (0.5%) or higher (1.5%) PAA concentrations may lead to incomplete surface coverage or excessive capping, which can result in particle agglomeration or suppressed growth, which hinder ion diffusion and electron transport. Variations in CV responses across the samples were directly influenced by the morphology and surface characteristics modulated by PAA content during synthesis [31,32]. Among the series, the CO-1 electrode demonstrated superior electrochemical behavior, as evidenced by its enhanced redox peak currents and larger enclosed CV area. This enhanced performance was attributed to the well-organized nanosheet architecture, which optimally balances high surface area and structural voids, thereby promoting rapid ion diffusion and accessible active sites. CV measurements at different scan rates revealed minimal distortion in the curve shape with the increasing scan rate (Figure 5b–d), indicating robust electrochemical reversibility and stable redox kinetics. The modest shifts in redox peak positions reflected increased electrode polarization at higher sweep rates, yet the overall retention of the profile shape affirmed efficient ion transport and consistent Faradaic behavior. The progressive enlargement of the CV area with the scan rate further confirmed improved charge diffusion and high utilization of redox-active surface sites. The distinct advantage of the CO-1 electrode over CO-0.5 and CO-1.5 was embedded in the precise tuning of morphology through surfactant control. The lower PAA concentration (0.5%) yielded less-defined structures with a reduced electroactive surface area, while the excessive concentration (1.5%) resulted in surface passivation due to over-capping, limiting ion accessibility. In contrast, the 1% PAA formulation promoted the formation of interconnected nanosheets arranged in an open porous network, providing sufficient channels for electrolyte infiltration and electron/ion mobility.

**Figure 6:**
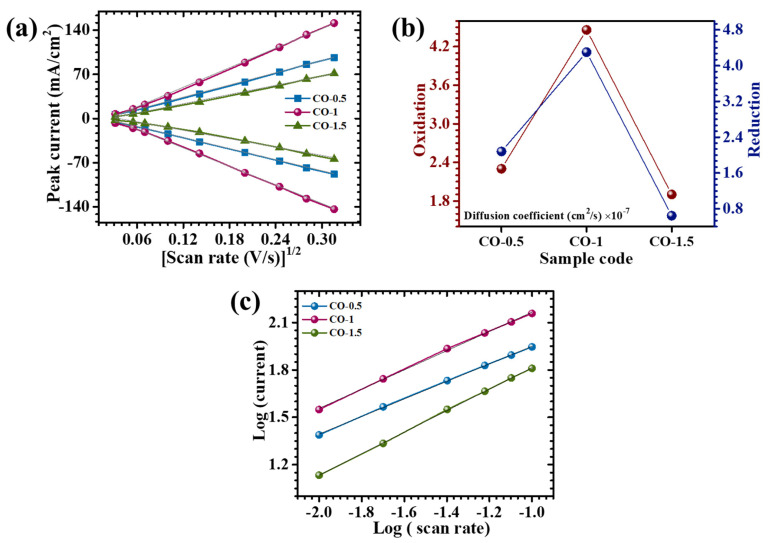
(**a**) Plot of peak current vs. (scan rate)^1/2^, (**b**) graphical representation of the calculated diffusion coefficient, and (**c**) plot of log(*i*) against the log(*ϑ*).

To gain deeper insights into the redox kinetics and ion transport dynamics of PAA-modified CO electrodes, CV analyses were performed across a series of scan rates. As illustrated in Figure 6a, a clear linear relationship between the anodic/cathodic peak current (*i_p_*) and the square root of the scan rate (*v*^1/2^) was observed for all electrode variants. This proportionality indicated that the electrochemical behavior was predominantly governed by diffusion-controlled processes, characteristic of reversible Faradaic reactions. To further quantify ion diffusion behavior, the apparent diffusion coefficients (*D*) were calculated using the Randles–Sevcik Equation (2) [33]:

(2) $$D^{1/2}=\frac{i_{p}}{2.69\times 10^{5}\times n^{3/2}\times A\times C\times v^{1/2}}$$

where *n* denotes the number of electrons involved in the redox process, *A* is the electrochemically active surface area, *C* represents the concentration of electroactive species in the electrolyte, and *v* is the scan rate. The calculated diffusion coefficients at a scan rate of 10 mV/s are compiled in Table 1, with a comparative graphical representation provided in Figure 6b. Among all studied electrodes, the CO-1 sample demonstrated the highest diffusion coefficient, indicative of enhanced ionic mobility and more efficient charge transfer kinetics. This improvement was ascribed to the optimized nanosheet-like architecture enabled by the incorporation of 0.1% PAA during synthesis. Conversely, both the CO-0.5 and CO-1.5 electrodes exhibited relatively lower diffusion coefficients. The inferior diffusion behavior of CO-0.5 was attributed to an underdeveloped nanostructure with limited surface accessibility, resulting from insufficient surfactant content. In the case of CO-1.5, excessive PAA likely induced excessive surface coverage or agglomeration, reducing the availability of electroactive sites and impeding ion diffusion pathways [34].

The charge storage behavior of the electrodes was systematically investigated by employing the power law equation ($i=av^{b}$), where *i* represents the peak current and *v* is the applied scan rate. The exponent *b* serves as a mechanistic indicator, offering insight into the predominant charge storage pathway. A *b*-value approximating 0.5 denotes a diffusion-limited Faradaic process, while *a* value approaching 1.0 suggests surface-dominated capacitive behavior [35]. As shown in Figure 6c, *b*-values were derived from the linear fit of log(*i*) versus log(*v*) plots. The extracted b-values for the CO series ranged from 0.55 to 0.68, as presented in Table 1. These values confirmed that the electrochemical processes in all electrodes were primarily governed by diffusion-controlled redox reactions, although capacitive contributions were not entirely negligible. To further define the relative contributions of surface-confined capacitive and diffusion-mediated processes, the current response at a slow scan rate was deconvoluted using the following expression [36]:

(3) $$i\left(V\right)=k_{1}v+k_{2}v^{1/2}$$

**Figure 7:**
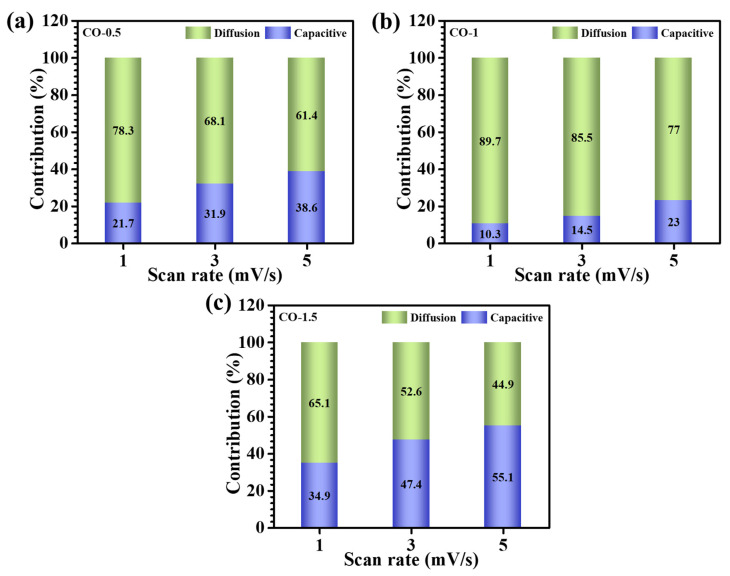
Capacitive and diffusion-controlled processes at different scan rates (1–5 mV/s): (**a**) CO-0.5, (**b**) CO-1, and (**c**) CO-1.5.

In this equation, *k*_1_*v* corresponds to the capacitive component, while *k*_2_*v*^1/2^ denotes the diffusion-controlled current. The constants *k*_1_ and *k*_2_ were obtained through linear regression of plots of *i*(*V*)/*v*^1/2^ vs. *v*^1/2^, allowing for quantification of the charge contributions. The total accumulated charge (*Q_t_*) within the CV profile could thus be partitioned into:

(4) $$Q_{t}=Q_{s}+Q_{d}$$

where *Q_s_* and *Q_d_* represent the surface-controlled and diffusion-controlled charges, respectively. As illustrated in Figure 7b, the CO-1 electrode demonstrated the highest diffusion-derived contribution, approximately 89.7% at 1 mV/s, highlighting its superior ionic diffusion kinetics. This was directly attributed to its well-engineered architecture consisting of interconnected nanosheets, which not only promoted facile electrolyte penetration but also provided extensive electroactive interfaces. The dependence of the charge storage mechanism on the scan rate was assessed over the slow scan range of 1–5 mV/s. As presented in Figure 7a–c, increasing the scan rate led to a progressive enhancement in the capacitive contribution across all samples. This trend was ascribed to the reduced electrolyte-ion residence time at higher sweep speeds, which restricted ion diffusion into the electrode bulk and thus favored near-surface charge accumulation [37,38].

**Figure 8:**
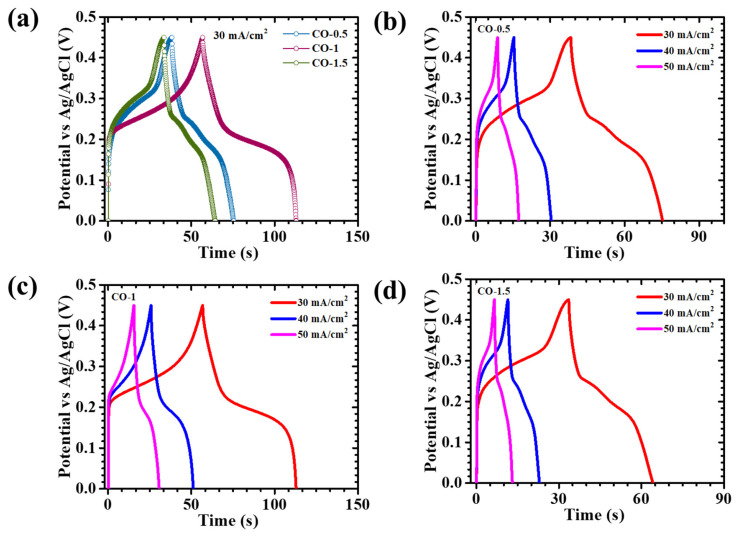
GCD curves of (**a**) CO electrodes at 30 mA/cm^2^ current density, and GCD plots of (**b**) CO-0.5, (**c**) CO-1, and (**d**) CO-1.5 electrodes at different current densities.

The GCD behavior of all CO-based electrodes was rigorously assessed at a constant current density of 30 mA/cm^2^ within the potential window of 0–0.45 V, as presented in Figure 8a. To further investigate the rate capability and charge storage dynamics, additional GCD measurements were performed over a current density range of 30 to 50 mA/cm^2^ for both PAA-modified (Figure 8b–d) and pristine CO (Figure S1b) electrodes. The observed discharge curves deviated significantly from the linear, triangular profiles typically associated with ideal capacitive behavior, thereby indicating the predominance of Faradaic redox processes. This characteristic profile is indicative of pseudocapacitive charge storage, wherein reversible redox reactions significantly contribute to overall capacitance. To accurately quantify electrochemical performance, the areal capacitance (C_A_), energy density (ED), and power density (PD) were calculated using integrated formulations that account for the nonlinear nature of the discharge curves, as presented in Equations (5)–(7) [39]:

(5) $$C_{A}=\frac{I\times 2\times \int V\left(t\right)dt}{A\times {\left(\Delta V\right)}^{2}}$$

(6) $$ED=\;\frac{1}{2\times 3600}\;C_{A}\times dV^{2}$$

(7) $$PD=\frac{ED\times 3600}{T_{d}}$$

**Figure 9:**
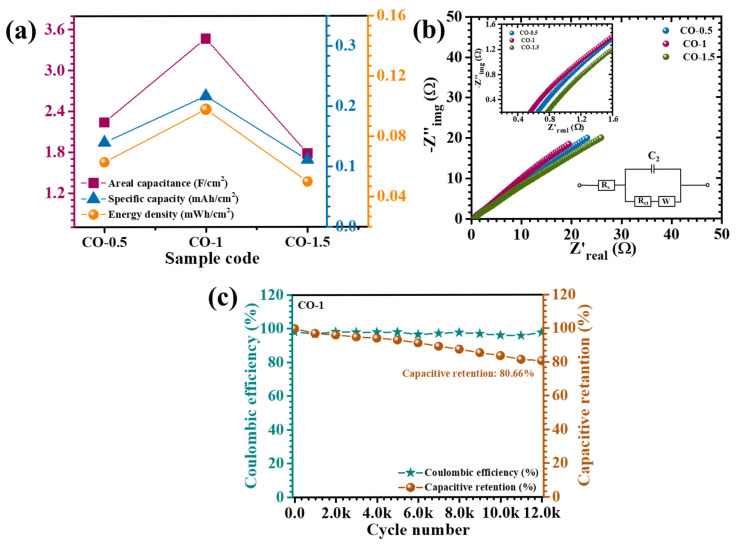
(**a**) Plot of calculated areal capacitance, (**b**) EIS analysis of CO electrodes (inset: EIS fitted circuit and enlarged EIS), and (**c**) cyclic stability over 12,000 GCD cycles of the CO-1 sample.

In these expressions, *I* is the applied discharge current, *∫V*(*t*)*dt* is the integration of the discharge curve in order to accurately calculate the capacitance due to the nonlinear GCD profiles, *A* is the electrode area, and Δ*V* is the applied voltage window. This integrated approach provides a more rigorous estimation of capacitance for pseudocapacitive systems, where charge storage is governed by reversible Faradaic processes and the GCD curves deviate from ideal linearity. Additionally, CV was used as an independent technique to validate the GCD-derived capacitance values. The areal capacitance from CV curves was calculated using the following relation (8) [39]:

(8) $$C_{A}=\frac{1}{\nu \times \Delta V\times A}\int I\left(V\right)dV$$

where *∫I*(*V*)*dV* is the area inside the CV curve, *I* is the current density as a function of voltage, *ν* is the scan rate, and *V* is the voltage window. The CV-derived capacitance values at a scan rate of 10 mV/s are shown in Table S1. The calculated areal capacitances for CO-0.5, CO-1, and CO-1.5 electrodes from GCD data at 30 mA/cm^2^ were 2237, 3467, and 1778 mF/cm^2^, respectively, while the pristine CO sample exhibited a significantly lower capacitance of 741 mF/cm^2^. These results, summarized in Table 2, also presented graphically in Figure 9a, offer a comparative framework for evaluating the electrochemical efficiencies of the various electrode formulations. The integration-based analysis of areal capacitance derived from both GCD and CV revealed a coherent and scientifically validated assessment of the electrochemical behavior of the PAA-modified Co_3_O_4_ electrodes. Despite employing different measurement techniques, both GCD and CV methods exhibited a consistent trend, wherein the CO-1 electrode demonstrated the highest areal capacitance across all current densities and scan rates. This alignment strongly supports the optimized structural configuration and superior charge storage capability of the CO-1 sample. The quantitative values obtained from CV were slightly different than those derived from GCD. This difference is justifiable, as CV captures dynamic redox behavior and transient current responses, often resulting in diverse capacitance values, especially at low scan rates where capacitive and Faradaic contributions overlap. In contrast, GCD measurements reflect time-integrated voltage responses and are more conservative in estimating capacitance under constant current discharge. The consistency in the performance trend across both CV and GCD supports the reliability of the electrochemical evaluation. The comparative data validated the pseudocapacitive nature of the electrodes and affirmed that the CO-1 sample, with its optimized nanosheet morphology, offers superior electrochemical characteristics. The presence of PAA acted as a structure-directing agent, modulating nucleation kinetics and promoting the growth of interconnected nanosheet networks. These nanosheets offer a high surface-to-volume ratio, open porosity, and accessible electroactive sites, which collectively facilitated rapid ion diffusion and efficient electrolyte penetration, while also supporting mechanical stability during charge–discharge cycling. Additionally, the nonlinear GCD profiles, particularly the initial voltage drop observed at the onset of discharge, reflected a rapid reduction in internal resistance. This phenomenon is indicative of efficient charge transfer at the electrode–electrolyte interface and low series resistance, which were both attributed to the conductive and structurally optimized nature of the CO-1 nanosheet framework [40].

EIS is an essential analytical technique for probing the charge transfer dynamics and resistive components of electrode systems. The Nyquist plots obtained for the CO electrodes, as shown in Figure 9b, exhibited a characteristic response consisting of a semicircular arc in the high-frequency region, transitioning into a linear segment at lower frequencies. The high-frequency intercept on the real axis (Z′) corresponds to the equivalent series resistance (ESR), which encompasses the collective contributions from the ionic resistance of the electrolyte, the intrinsic resistance of the electrode material, and the interfacial contact resistance between the electrode and the electrolyte [41]. To gain a comprehensive understanding of the interfacial charge transfer and ion diffusion characteristics of the PAA-assisted CO electrodes, the Nyquist plots were fitted using an appropriate equivalent circuit model (inset of Figure 9b). The extracted fitting parameters are presented in Table 1. Notably, the CO-1 electrode exhibited the lowest charge transfer resistance (R_ct_ = 0.4267 Ω) and the smallest solution resistance (R_s_ = 1.21 Ω), indicative of superior electronic conductivity and enhanced charge transport at the electrode–electrolyte interface. This significant reduction in resistance was directly attributed to the uniform and well-structured nanosheet architecture achieved via optimal PAA incorporation during synthesis. The finely tuned morphology facilitated efficient ion diffusion and rapid electron transfer, thereby promoting improved electrochemical response. In comparison, the CO-0.5 and CO-1.5 electrodes demonstrated higher R_s_ and associated impedance values, reflecting hindered ion transport and interfacial resistance. These limitations were likely due to suboptimal microstructural features, such as irregular growth or partial agglomeration, which impeded active site accessibility and ion conduction.

To further evaluate the electrochemical durability, the cycling performance of the CO-1 electrode was assessed over 12,000 continuous charge–discharge cycles at a high current density of 70 mA/cm^2^ (Figure 9c). Impressively, the electrode maintained 80.66% of its initial capacitance with minimal deterioration, equivalent to a capacitance loss of only ~19.3%, and retained a high coulombic efficiency of 97.4%. These results demonstrate the excellent long-term stability and reversibility of the charge storage mechanism. The superior cycling retention was primarily attributed to the well-defined nanostructure induced by the optimal PAA concentration, which directed the formation of interconnected porous nanosheets. This architecture not only buffered mechanical stress during repeated redox reactions but also promoted efficient electrolyte penetration and facilitated rapid ion diffusion throughout the active matrix. The structural integrity of the CO-1 electrode thus remained largely intact even under prolonged electrochemical stress. The slight performance attenuation observed during extended cycling was likely due to progressive ion entrapment phenomena. Over time, ions may become immobilized within confined pores or accumulate at the electrode–electrolyte interface, thereby reducing the availability of electroactive sites for effective charge exchange. This phenomenon gradually limits the reversible Faradaic activity and results in a moderate decline in capacitance [7]. Overall, the findings underscore the critical importance of morphology control and surface engineering in dictating the electrochemical behavior of pseudocapacitive materials.

A comparative evaluation of the electrochemical performance of the CO-1 electrode with previously reported Co_3_O_4_-based systems is summarized in Table S2 [42,43,44,45,46,47,48]. Most existing studies reported areal capacitance values below 3000 mF/cm^2^, typically achieved at low current densities and lower cycling stabilities. In contrast, the current electrode achieved a significantly higher areal capacitance of 3467 F/cm^2^ at 30 mA/cm^2^, along with excellent cycling stability of 12,000 cycles. Importantly, this performance was attained using a simple, binder-free electrodeposition method without high-temperature treatment or complex composite architectures. These results highlight the superiority and practicality of the PAA-assisted synthesis strategy, offering a scalable and effective route for developing high-performance pseudocapacitive electrodes.

A relative FE-SEM analysis was conducted after 12,000 GCD cycles, as shown in Figure S2a,b. Importantly, post-cycling FE-SEM images confirmed that the nanosheet framework was largely retained, with no observable collapse, aggregation, or severe deformation. The layered morphology remained accessible and interconnected, indicating excellent structural resilience under electrochemical stress. This observation was in direct agreement with the electrochemical performance metrics that strongly reflected mechanical and chemical stability. While minor surface roughening and edge thinning were evident, likely resulting from repeated ion insertion/extraction processes, a majority of the nanosheets remained structurally intact, demonstrating only minimal degradation.

To validate the pseudocapacitive charge storage mechanism involving multiple cobalt oxidation states, high-resolution XPS analysis was conducted on the CO-1 electrode after electrochemical cycling. The Co 2p spectrum (Figure S2c) showed distinct peaks corresponding to Co 2p_3/2_ and Co 2p_1/2_ centered around ~779.7 eV and ~795.2 eV, respectively. The deconvolution of the Co 2p_3/2_ region revealed the presence of both Co^2+^ and Co^3+^ species, confirmed by characteristic peaks. Additionally, a subtle shoulder at a slightly higher binding energy suggested the emergence of Co^4+^ species, which is indicative of progressive oxidation during repeated redox cycling. This observation aligns with literature reports showing that Co^3+^ can further oxidize to Co^4+^ under alkaline conditions. The accompanying O 1s spectrum (Figure S2d) further supports this analysis. It showed a dominant peak at ~529.8 eV attributed to lattice oxygen (O^2−^), and a secondary peak near ~531.2 eV corresponding to surface hydroxyl groups (–OH), which were actively involved in the reversible insertion/extraction of OH^−^ ions during cycling, as detected by the increased area of the secondary peak. Further, the persistence and relative stability of these peaks confirmed the retention of the electroactive oxide framework and the continued accessibility of redox-active sites. When compared to the pre-cycling XPS spectra, the relative increase in Co^3+^/Co^4+^ contributions and the consistent O 1s profile suggest that the Co_3_O_4_ electrode underwent a reversible multivalent redox process, which is essential for its pseudocapacitive behavior [49,50].

## 6. Electrochemical Performance of Asymmetric Supercapacitor Device

**Figure 10:**
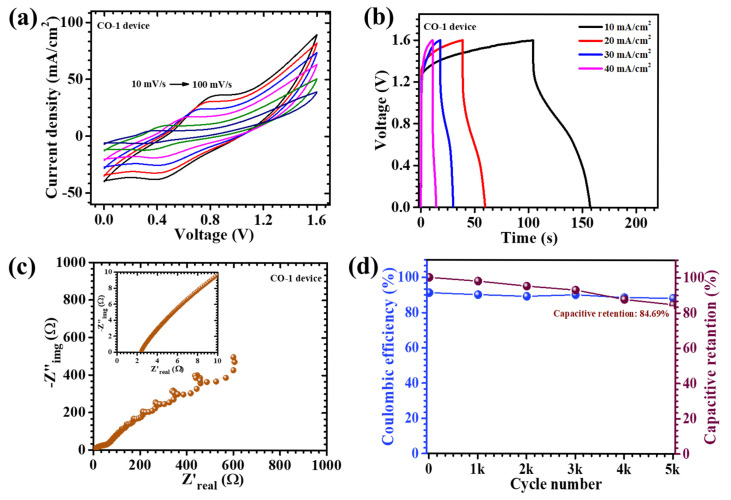
(**a**) CV tests performed on the CO-1//AC device recorded at a scan rate of 10–100 mV/s across a potential range of 0 to 1.6 V, (**b**) GCD measurements at different current densities for the CO-1//AC device, (**c**) EIS measurement of the device, and (**d**) cyclic stability of 5000 GCD cycles of the CO-1//AC device.

To evaluate the real-world applicability of the synthesized CO electrode, an asymmetric pouch-type supercapacitor device (APSD) was fabricated and rigorously characterized. In this device architecture, the CO-1 sample functioned as the pseudocapacitive positive electrode, while activated carbon (AC), known for its electric double-layer capacitance behavior, served as the negative electrode. Both materials were uniformly deposited onto nickel foam substrates to ensure optimal electrical contact. A 2 M KOH aqueous electrolyte, absorbed into filter paper, was employed as the ionic medium and separator, while the entire device was sealed to mitigate environmental interference and moisture ingress. The electrochemical properties of the APSD were systematically investigated via CV, GCD, and EIS. The CV profiles (Figure 10a), recorded over a range of scan rates from 10 to 100 mV/s, exhibited non-rectangular shapes with a progressively enhanced current response, reflecting fast ion diffusion and good electrochemical reversibility. Notably, the device maintained a stable potential window up to 1.6 V, which is exceptionally broad for aqueous-based systems and indicative of enhanced electrochemical kinetics. This extended operating range resulted from the effective synergy between the high redox activity of the CO electrode and the high surface area of the AC electrode, collectively promoting superior charge storage characteristics. The GCD measurements (Figure 10b) further corroborated the capacitive nature of the device. The discharge profiles revealed typical pseudocapacitive features, including nonlinear characteristics, suggestive of Faradaic processes. At a current density of 10 mA/cm^2^, the device achieved a remarkable areal capacitance of 157 mF/cm^2^, an energy density of 0.056 mWh/cm^2^, and a power density of 1.9 mW/cm^2^ (Table 3).These values signify the device’s capability to concurrently offer high energy and power output, rendering it suitable for high-performance energy storage applications. EIS measurements, shown in Figure 10c, provided additional insight into the internal resistive components of the device. The Nyquist plot featured a semicircle in the high-frequency domain and a linear segment in the low-frequency region. The derived equivalent series resistance (ESR) was as low as 2.43, indicative of efficient ion/electron transport at the electrode–electrolyte interface. This low resistance can be attributed to the well-optimized nanostructure of the CO-1 electrode, which ensured continuous electron pathways and facile electrolyte infiltration. To assess long-term electrochemical durability, the APSD underwent prolonged charge–discharge cycling for 5000 consecutive cycles at a high current density of 60 mA/cm^2^ (Figure 10d). Impressively, the device retained 84.69% of its initial capacitance, coupled with a high coulombic efficiency of 88.41%, reflecting excellent charge retention and minimal capacity declining. The sustained performance resulted from the mechanically robust and hierarchically porous nanosheet architecture of the CO-1 electrode, which accommodated structural strain during repeated cycling and prevented degradation. In summary, the APSD demonstrated a compelling combination of high areal capacitance, an extended operational voltage window, low internal resistance, and remarkable cycling stability. These attributes collectively underscore the potential of the CO-1 electrode as a viable candidate for integration into next-generation flexible and portable supercapacitor platforms. The findings further emphasize the significance of surfactant-assisted synthetic strategies in engineering advanced electrode architectures tailored for high-performance energy storage applications [51,52].

## 7. Conclusions

In conclusion, this study presented an effective and scalable approach to fabricate high-performance CO electrodes via PAA-assisted electrodeposition. The optimized CO-1 sample (1% PAA) delivered outstanding electrochemical performance, including a high areal capacitance of 3467 mF/cm^2^, low charge transfer resistance (0.4267 Ω), and a dominant diffusion-controlled charge storage contribution (91.7%). The tailored interconnected nanosheet architecture enabled enhanced ion transport and efficient redox activity. To demonstrate practical application, an asymmetric pouch-type supercapacitor was assembled using CO-1 and activated carbon as electrodes. The device exhibited stable operation within 1.6 V, achieving an areal capacitance of 157 mF/cm^2^, an energy density of 0.056 mWh/cm^2^, and a power density of 1.9 mW/cm^2^, with excellent cycling stability. Overall, this work underscores the significance of surfactant-assisted electrode design for improving energy storage materials and offers a promising strategy for developing next-generation flexible and portable supercapacitor devices.

## Figures and Tables

**Table 1:** Estimated diffusion coefficient, b-values, and series resistance values of CO-0.5, CO-1, and CO-1.5 samples.

Sample Code	Diffusion Coefficient (cm^2^/s) × 10^−7^		*b*-Value	R_s_ (Ω)	R_ct_ (Ω)
	Oxidation	Reduction			
**CO-0.5**	2.3	2.08	0.6	0.5342	1.84
**CO-1**	4.46	4.3	0.55	0.4267	1.21
**CO-1.5**	1.9	0.65	0.68	0.6703	3.52

**Table 2:** Evaluation of calculated areal capacitance, specific capacity, energy density, and power density values of CO, CO-0.5, CO-1, and CO-1.5 electrodes from GCD measurements.

Sample Code	I (mA)	C_A_ (mF/cm^2^)	C (mAh/cm^2^)	ED (mWh/cm^2^)	PD (mW/cm^2^)
**CO**	30	741	0.046	0.021	5.21
	40	632	0.040	0.018	6.74
	50	543	0.034	0.015	8.09
**CO-0.5**	30	2237	0.140	0.063	6.22
	40	1185	0.074	0.033	7.89
	50	840	0.052	0.024	10.00
**CO-1**	30	3467	0.217	0.098	6.27
	40	1936	0.121	0.054	7.84
	50	1383	0.086	0.039	9.21
**CO-1.5**	30	1778	0.111	0.050	5.90
	40	909	0.057	0.026	8.07
	50	642	0.040	0.018	10.16

**Table 3:** Calculated energy storage parameters of the CO-1//AC asymmetric pouch-type supercapacitor device.

Sample Code	I (mA)	C_A_ (mF/cm^2^)	C (mAh/cm^2^)	ED (mWh/cm^2^)	PD (mW/cm^2^)
**CO-1 device**	10	157	0.035	0.056	1.90
	20	101	0.022	0.036	3.01
	30	100	0.022	0.035	4.90
	40	23	0.005	0.008	4.05

## Data Availability

The original contributions presented in the study are included in the article/Supplementary Materials, further inquiries can be directed to the corresponding author.

## Supplementary Materials

The following supporting information can be downloaded at: https://www.mdpi.com/article/10.3390/ma18122916/s1, Figure S1: (a) CV tests performed on the CO electrode recorded at a scan rate of 10–100 mV/s across a potential range of 0 to 0.5 V, (b) GCD measurements at different current densities for CO electrode; Figure S2: (a,b) FESEM images and (c,d) XPS analysis of CO-1 electrode after long-term cycling stability; Table S1: Evaluation of calculated areal capacitance CO, CO-0.5, CO-1, and CO-1.5 electrodes from CV measurements; Table S2: Energy storage parameters comparison of the current study with the existing literatures.
