# Supplementary material for: Polyacrylic Surfactant-Enabled Engineering of Co3O4 Electrodes for Enhanced Asymmetric Supercapacitor Performance
Source: Materials (Basel). 2025 Jun 19;18(12):2916. doi: 10.3390/ma18122916 (PMC12195595; doi:10.3390/ma18122916)
Supplement: Supplementary file 1 [file materials-18-02916-s001.zip › materials-3609854-supplementary.pdf]

Supplementary File

Figure S1: (a) CV tests performed on the CO electrode recorded at a scan rate of 10-100 mV/s across a potential range of 0 to 0.5 V, (b) GCD measurements at different current densities for CO electrode.

Figure S2: (a, b) FESEM images and (c, d) XPS analysis of CO-1 electrode after long-term cycling stability.

Table S1: Evaluation of calculated areal capacitance CO, CO-0.5, CO-1, and CO-1.5 electrodes from CV measurements.

Table S2: Energy storage parameters comparison of the current study with the existing literatures.

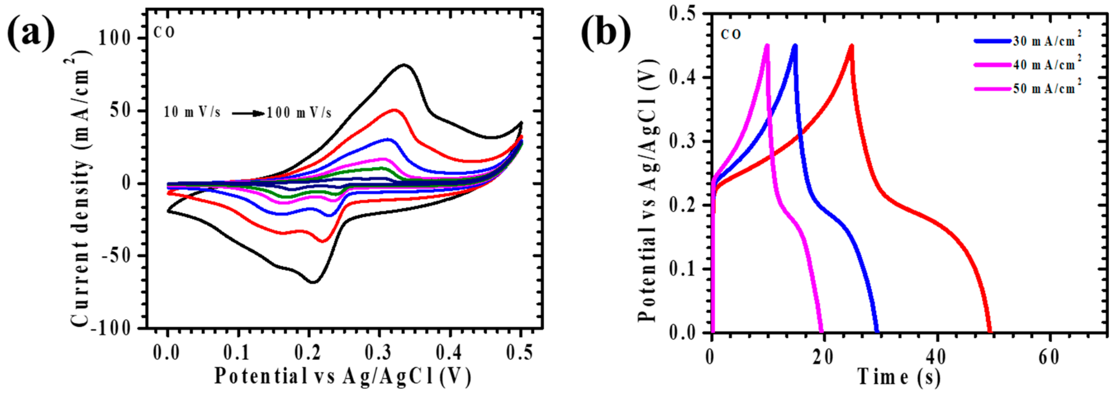

Figure S1 (a, b)

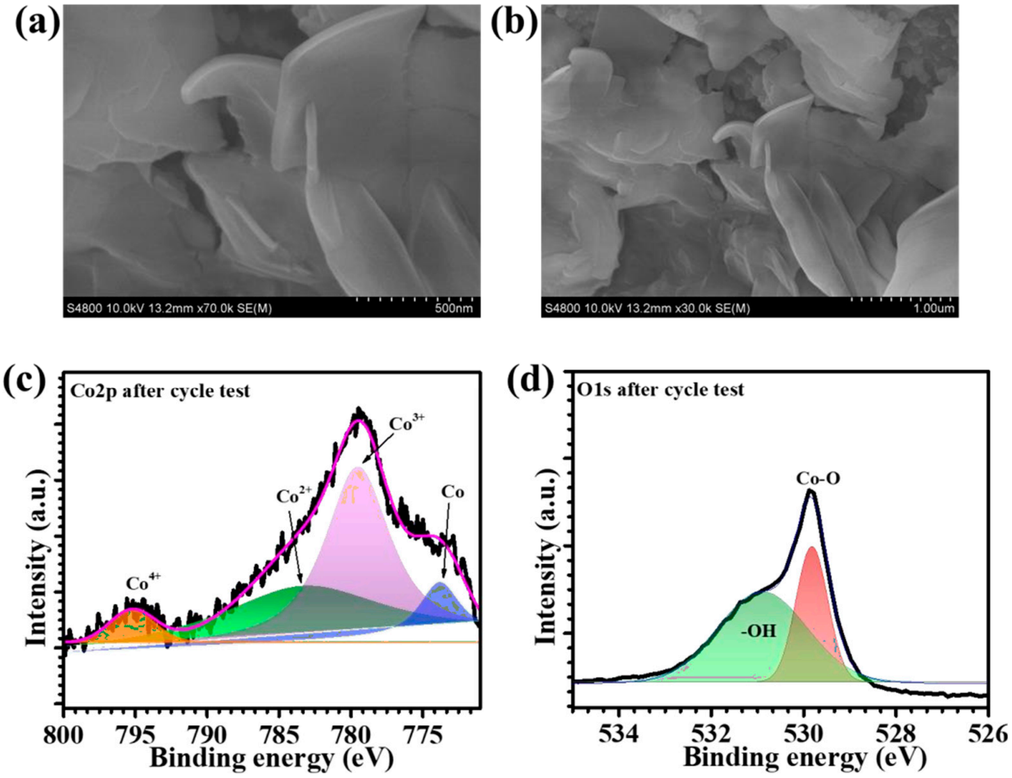

Figure S2 (a-d)

Sample Code	Scan Rate (mV/s)	CA (mF/cm^2)
CO-0.5	10	900
CO-1	10	1100
CO-1.5	10	340

Table S1

Sr. No.	Material	Synthesis Method	Current (mA/ cm^2)	Areal Capacitance (mF/cm^2)	Cycle Stability	Reference
1.	CeO_2@Co_3O_4	Hydrothermal	1	1598	6000 cycles (96.6% stability)	42
2.	Co_3S_4/NiS	Hydrothermal	4	1810	2400 cycles	43
3.	Co_3O_4@Au@CuO core–shell	Au sputtering	4.8	240	10,000 cycles	44
4.	Co_3O_4/Ni(OH)$_2$	Hydrothermal	5	2665.76	5000 cycles	45
5.	Mo-Co_3O_4@NF	Solvothermal	1	2815	5000 cycles (90% stability)	46
6.	Co_3O_4@Co-CH	Solvothermal	1	1541	5000 cycles (72.1% stability)	47
7.	Co_3O_4/VGN hybrid	Pulsed laser deposition	-	32	3000 cycles (89% stability)	48
**8.**	**PAA-Co_3O_4**	**Electrodeposition**	**30**	**185.25**	**12, 000 cycles (80.66% stability)**	**This work**

Table S2
